# Correlation between temporal muscle thickness and grip strength in hemiplegic patients with acute stroke

**DOI:** 10.3389/fneur.2023.1252707

**Published:** 2023-11-23

**Authors:** Jisoo Park, Jihye Park, Soye Kim, Dong Chan Kim

**Affiliations:** Department of Rehabilitation Medicine, Eunpyeong St. Mary's Hospital, College of Medicine, The Catholic University of Korea, Seoul, Republic of Korea

**Keywords:** sarcopenia, ischemic stroke, grip strength, temporal muscle thickness, hemiplegia after stroke

## Abstract

Recently, temporal muscle thickness (TMT) has been investigated as a novel surrogate marker for muscle mass and function in neurologic patients. This study aimed to assess the correlation of TMT with grip strength to establish a new parameter for predicting pre-stroke sarcopenia. A total of 358 patients who were newly diagnosed with acute ischemic stroke at our institution between November 2021 and August 2022 were enrolled. Eighty-four patients met the eligibility criteria. The mean TMT was measured within initial brain MRI using previously described methods. Pearson's correlation analyses assessed the relationship between grip strength and TMT. Multiple logistic regression analyses were performed to identify associations between TMT and other associated factors including grip strength, sarcopenia risk, body mass index, age, Charlson Comorbidity Index and Geriatric nutrition risk index. Mean TMT values indicated a strong correlation with the grip strength of the non-hemiplegic hand in both male and female patients. Multiple logistic regression analyses showed that TMT was associated with grip strength and sarcopenia risk in hemiplegic patients. Measuring TMT using cranial MR images during the initial stages of stroke could help predict a patient's pre-stroke muscle strength status. Further studies are required to apply TMT in pre-stroke sarcopenia diagnosis.

## 1 Introduction

Sarcopenia has been defined as a progressive and generalized skeletal muscle disorder that involves the accelerated loss of muscle mass and function (1). The incidence of sarcopenia in the Asian population in previous studies indicates that its prevalence is quite high; 13.1–14.9% in men and 11.4% in women aged 65 years old or older in the Korean population, 11.5% in men and 16.7% in women in 65 years old or older in the Japanese population and 18.6% in women aged 60 years old or older in the Chinese population (2–4). It is widely agreed that sarcopenia could lead to mobility impairment, disability, loss of independence, hospitalization, and death (1). Stroke is one factor that is closely related to sarcopenia. Pre-stroke sarcopenia is associated with poor functional outcomes after stroke (5). The diagnosis of sarcopenia, which is suggested by the European Working Group on Sarcopenia in Older People and the Asian Working Group for Sarcopenia (AWGS), includes the evaluation of muscle strength, physical performance, and the appendicular skeletal muscle mass (1, 6).

Physical frailty is a subset of frailty characterized by the frailty phenotype involving unintentional weight loss, self-reported exhaustion, weakness which is substituted as low grip strength, slow walking speed, and low physical activity (7). Physical frailty and sarcopenia are closely related and sarcopenia has been described as the biological substrate of physical frailty (8). As in sarcopenia, frailty also is closely related to stroke. One group suggested that almost 80% of the patients admitted to an acute stroke unit were either frail or pre-frail according to Frailty Index criteria (9). Correlation between pre-stroke frailty and stroke severity was also previously reported (10). Importance of the diagnosis of frailty and sarcopenia in acute stroke patients is noted.

Among various measurements, hand grip strength is widely used in measuring maximal voluntary muscle strength. Hand grip strength is correlated with the patient's general nutritional status (11, 12) and medical condition. Grip strength on the unaffected side in acute stroke patients with hemiparesis certainly plays a significant role in sarcopenia evaluation; it not only represents the overall muscle strength (13), but is also suggested as an independent predictor of the short-term post stroke functional outcome (14). However, a crucial requirement in this evaluation is the patient's cooperation, which may not always be possible in acute stroke patients, due to cognitive decline or sedation.

One effective tool for measuring skeletal muscle mass is dual-energy x-ray absorptiometry (DXA) (15). However, additional costs are required to routinely apply it to stroke patients in routine clinical practice. As previous studies demonstrated a high correlation between the skeletal muscle mass (which was assessed by DXA) and the skeletal muscle cross-sectional area at the level of the third lumbar vertebra obtained by CT images, abdominal CT is now proposed as a tool for muscle mass measurement (1, 16). However, it requires additional radiation exposure to patients as abdominal imaging is not required in the routine diagnosis of stroke. Several techniques have been used to estimate muscle mass; however, all of them have major limitations, including variability in their results, the inconsistent use of cutoff points, and the weak association between muscle mass and adverse health outcomes (17).

Recently, temporal muscle thickness (TMT) measured on brain CT or magnetic resonance imaging (MRI) has been introduced as a novel surrogate marker and sarcopenia predictor (18). One study showed TMT and grip strength were highly correlated in healthy individuals (19). Currently, multiple studies suggest TMT as a biomarker for skeletal muscle mass measurement and as a prognosis predictor in patients with brain tumors (20–25). However, there are only a few studies on TMT among patients with acute ischemic stroke.

As there are many limitations in diagnosis of pre-stroke sarcopenia, such as cognitive decline and extra-radiation exposure, we aimed to evaluate relevance of TMT as a new marker to predict pre-stroke sarcopenia. In this study, we hypothesized TMT measurement through brain MRI would have positive association with grip strength in acute ischemic stroke patients with unilateral hemiplegia.

## 2 Materials and methods

### 2.1 Participants

This retrospective study was approved by the Institutional Review Board of Eunpyeong Saint Mary's Hospital (IRB PC22RASI0242). The need for consent was waived by the ethics committee. After approval, the electronic medical records of newly–diagnosed stroke patients treated at one hospital between November 2021 and August 2022 were examined. The inclusion criteria were: (1) first ischemic stroke diagnosed via an initial brain MRI, (2) unilateral hemiplegia, (3) initial rehabilitation assessment (K-MBI, grip strength, Fugl-Meyer) performed within 1 week after admission, and (4) Fugl-Meyer score of non-hemiplegic hand scoring maximum score of 14 points, showing that the functional ability of the measured hand was intact. The exclusion criteria were: (1) history of previous stroke (*n* = 31), (2) cerebrovascular diseases other than ischemic stroke [traumatic brain injury (*n* = 36), subarachnoid hemorrhage (*n* = 26), intracranial hemorrhage (*n* = 49) etc.], (3) patients with incomplete data (initial body weight, height, and rehabilitation assessment regarding the first week after admission) (*n* = 131).

### 2.2 Data collection

Each patient's age, sex, stroke type, stroke lesion, hemiplegia side, height (cm), body weight (kg), hand grip strength of the non-hemiplegic side (kg), Fugl-Meyer score, SARC-F (strength, assistance with walking, rising from a chair, climbing stairs, and falls questionnaire) score, Korean version of the Modified Barthel Index (K-MBI), Charlson Comorbidity Index (CCI), and Geriatric nutrition risk index (GNRI) were recorded. Each patient's height and weight were used to calculate the body mass index (BMI: weight divided by height squared). The GNRI was calculated using the following formula: GNRI = [1.489 × serum albumin (g/dL)] + 103 [41.7 × weight (kg)/ideal body weight]. This index enables quantitative determination of the risk of nutrition-related morbidity and mortality in elderly patients at admission into a geriatric hospital (26). The CCI was used to evaluate patients' comorbidities. Each comorbidity category has an associated weight (from 1 to 6), based on the adjusted risk of mortality or resource use, and the sum of all the weights results in a single comorbidity score for a patient (27). SARC-F questionnaire is a screening tool for initial sarcopenia assessment. SARC-F scores ranged from 0 to 10 (0 = good, 10 = high possibility of sarcopenia).

### 2.3 Temporal muscle thickness measurement

**Figure 1:**
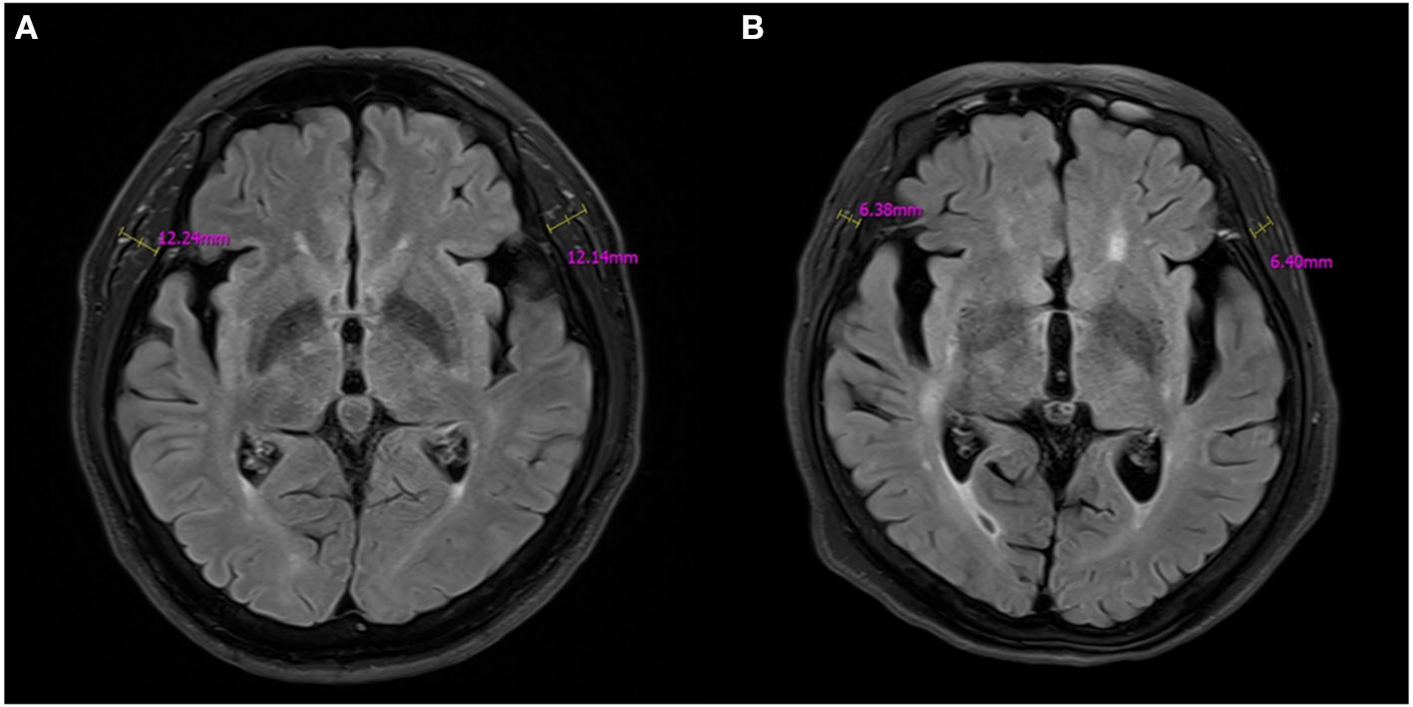
Examples of TMT measurements on MR FLAIR images. **(A)** A 67-year-old male patient with left basal ganglia infarction, **(B)** A 78-year-old female patient with left pontine infarction.

Brain MRI was performed with a 3-T MRI unit (MAGENETOM Verio, Siemens Healthcare, Erlangen, Germany). TMT was measured manually on the image in axial fluid-attenuated inversion-recovery (FLAIR, TR/TE/TI, 8000/114/2370 ms; section thickness: 4 mm; Field of view: 210 x 210 mm), which was routinely performed in our hospital for all patients diagnosed with ischemic stroke. Maximum TMT measurements were done at the level of the orbital roof, perpendicular to the long axis of the temporal muscle on both sides, as suggested in previous studies (19–25, 28–30). The pre-defined anatomical reference point regarding anterior–posterior orientation was the Sylvian fissure. Figure 1 shows examples of TMT measurement. The mean TMT of the right and left sides was used for the analysis. The first author manually investigated the TMT of 249 patients who underwent an initial brain MRI. After being blinded to the diagnosis, sex, age, and grip strength, the measurement was repeated to determine the intra-rater reliability. A second rater conducted the TMT measurement of 84 patients who were finally enrolled in the study after being blinded to the data to check the inter-rater reliability of the measurement.

### 2.4 Grip strength measurement

Hand grip strength was routinely measured in both hands within 1 week for every acute stroke patient in our hospital by an experienced occupational therapist using a Digital Hand Dynamometer (JAMAR, Patterson Medical, UK). We followed the hand grip strength measurement technique proposed in the AWGS 2019 guideline, which suggests taking the maximum reading of at least two trials using either both hands or the dominant hand in a maximum-effort isometric contraction, rather than using a fixed acquisition time. Since our study participants showed unilateral hemiplegia, the non-hemiplegic hand was selected regardless of whether or not it was the dominant hand. Patients were ordered to perform maximal contraction in the sitting position with 90 degrees of elbow flexion twice using the non-hemiplegic hand. The higher grip strength from these two trials was selected for the analysis. The rater was blinded to the measured TMT value.

### 2.5 Statistical analysis

We used SPSS Statistics for Windows (version 26; IBM Corp., Armonk, NY, USA) for all data analyses. The inter-rater reliability and intra-rater reliability of TMT were assessed using interclass correlation coefficient (ICC) estimates, and their 95% confidence intervals (CIs) based on mean-rating (k = 2), absolute-agreement, and the two-way mixed effects model (31). Pearson's correlation analysis was first done to assess whether the relationship between grip strength and TMT exists. Analyses were performed after stratifying by sex since grip strength and skeletal muscle components differ with sex. According to the AWGS guidelines, low grip strength is diagnosed as <28.0 kg for men and <18 kg for women. Also, a previous study reported a significant difference in TMT of healthy patients between males and females (19). Multiple linear regression analyses were then performed to assess the associations between TMT and grip strength and other multiple variables, including SARC-F, age, sex, BMI, CCI and GNRI. Multicollinearity was assessed using the Variance Inflation Factor (VIF). A VIF value of 1–10 was considered to indicate the absence of multicollinearity. As a secondary outcome, we derived the adjusted odds ratio of the initial functional status within a week after stroke and sarcopenia risk with the mean TMT via logistic regression. K-MBI scores ranged from 0 to 100 (0–24 = total dependency, 25–49 = severe dependency, 50–74 = moderate dependency, 75–90 = mild dependency, 91–99 = minimal dependency, 100 = independent). Patients with scores of <75 were classified as having poor initial functional status after stroke. Independent samples *T* test was performed to compare the days from stroke onset to K-MBI measurement between the two groups. Odds ratios were adjusted for age, sex, BMI, CCI, and GNRI. *P*-values of <0.05 were considered statistically significant. The power analysis was performed using G^*^power 3.1 (32).

## 3 Results

Among 358 patients who were diagnosed with acute stroke, 84 met the eligibility criteria. Patient demographics are presented in Table 1. Fifty-two patients (61.9%) were male. The patients' mean age was 69 ± 13.15 (33–91) years. The mean BMI was 23.4 ± 3.13 (15.43–32.05) kg/m^2^. Forty-one (48.8%) patients had insufficient grip strength according to cutoff points per the AWGS criteria. The mean TMT was 8.26 ± 1.28 mm (range: 5.40–11.01 mm) in females and 10.24 ± 1.43 mm (range: 7.34–12.71 mm) in males, which was similar to the values reported in previous Japanese study (28).

**Table 1:** Participants' demographics.

**Category**	**Total (*n* = 84)**	**Male (*n* = 52)**	**Female (*n* = 32)**
Age (years)	69 ± 13.15	64.5 ± 12.79	72.5 ± 13.36
BMI (kg/m^2^)	23.4 ± 3.13	23.4 ± 2.94	23.4 ± 3.40
Hemiplegic hand, right/left	51/33	35/17	16/16
Brain lesion, supratentorial/infratentorial	63/21	38/14	25/7
**K-MBI score on admission**			
0~24	23	9	14
25~49	24	17	7
50~74	19	14	5
75~90	16	10	6
91~99	2	2	0
GNRI	108.86 ± 8.52	108.35 ± 8.10	109.48 ± 9.11
**Comorbidities**			
CCI	5.5 ± 1.96	4.5 ± 2.07	6 ± 1.74
Hypertension	34	19	15
Diabetes mellitus	22	15	7
COPD	1	1	0
CKD	0	0	0
Mild cognitive impairment	2	0	2
Low hand grip strength^†^	41	18	23

^†^The values represent the mean ± SD or frequency (percentage).^†^Low hand grip strength per the AWGS guidelines; a hand grip strength of < 28.0 kg for men and < 18 kg for women.

**Figure 2:**
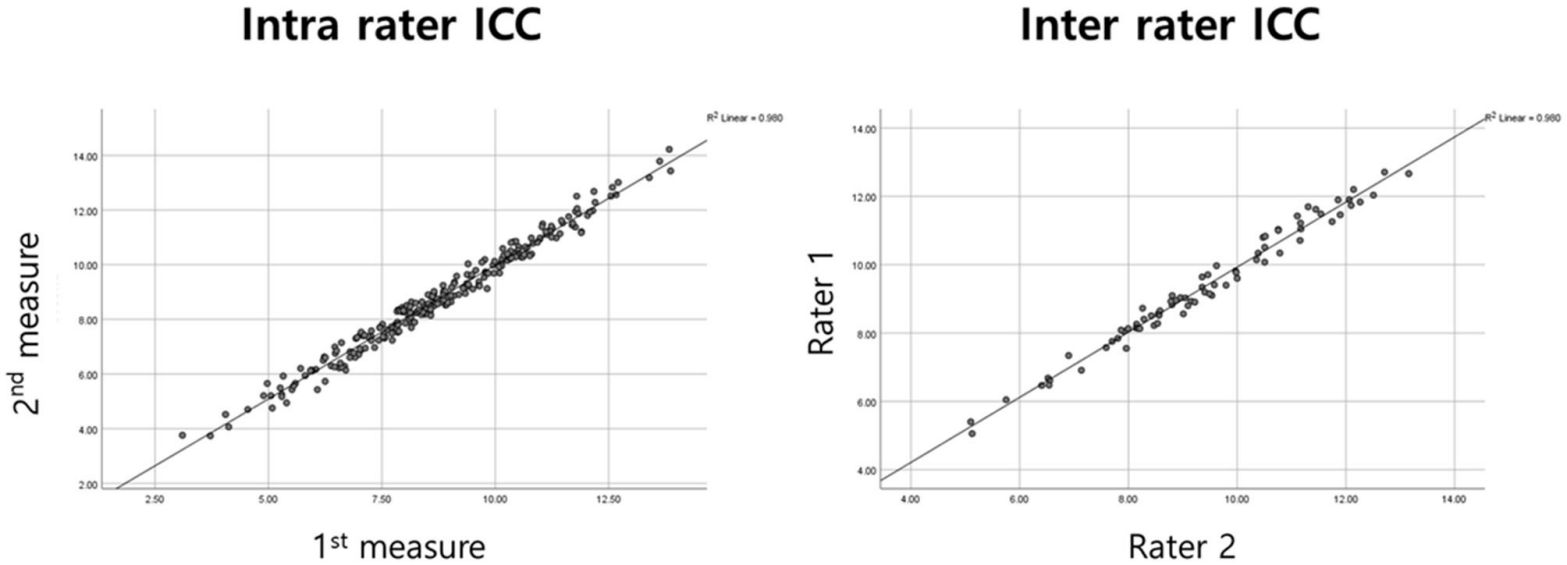
Intra-rater and Inter-rater reliability of TMT measurements. Interclass correlation coefficients (ICC) were analyzed to check reliability of TMT measurement method. Intra-rater ICC distributes agreements between single rater's 1^st^ measure (x-axis) and 2^nd^ measure (y-axis). Inter-rater ICC distributes agreements between rater 2 (x-axis) and rater 1 (y-axis).

Both inter-rater reliability [ICC = 0.99 (95% confidence interval: 0.987–0.992), *p*-value < 0.001] and intra-rater reliability [ICC = 0.989 (95% confidence interval: 0.983–0.993), *p*-value < 0.001] were excellant as reported in many other previous studies (Figure 2).

**Figure 3:**
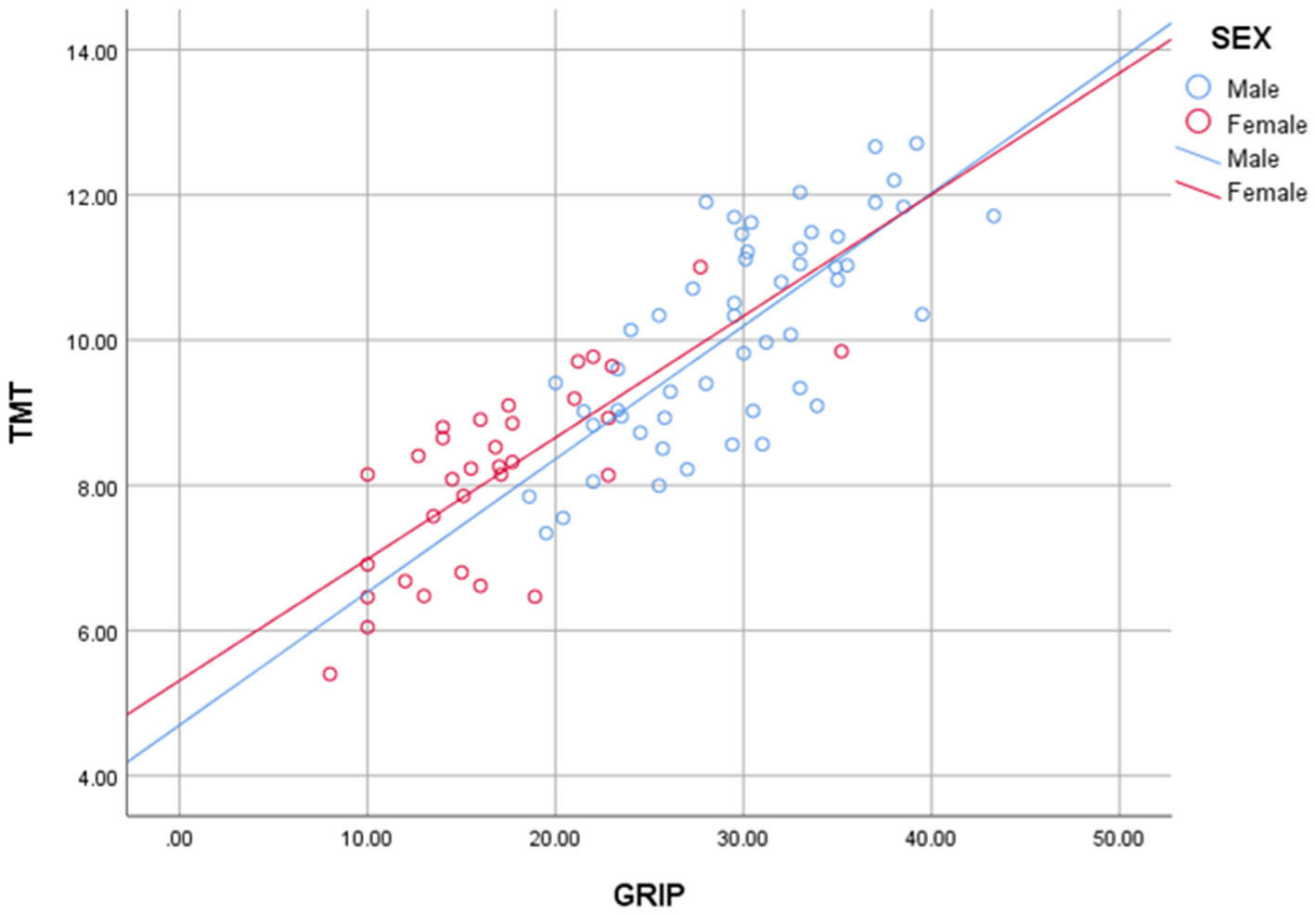
Scatter diagrams of grip strength vs. TMT. Male (blue) and female (red) patients' grip strength (x-axis) and TMT (y-axis) are distributed as dots with linear regression.

Our primary outcome was the existence of a correlation between TMT and grip strength. As shown in Figure 3, TMT values showed a strong correlation with the grip strength of the non-hemiplegic hand in both male (Pearson's correlation coefficient: 0.744; *p* < 0.001) and female (Pearson's correlation coefficient: 0.735; *p* < 0.001) patients. Multiple linear regression analyses revealed that the mean TMT was positively associated with grip strength (β = 0.634, *p* = 0.000) in hemiplegic stroke patients. SARC-F scores (β = −0.0.181, *p* = 0.012) and age (β = −0.0.200, *p* = 0.015) was negatively associated with the mean TMT. Also, the mean TMT differed statistically by sex (β = −0.0.425, *p* = 0.000). However, no significant correlation between TMT and other variables was observed, (*p*-values scoring > 0.05) in BMI, GNRI and CCI. There was no multicollinearity between the variables. *R*^2^ values of these models were higher than 0.4, showing that this is a highly-explanatory model (*R*^2^ = 0.737; Table 2).

**Table 2:** Multiple linear regression analysis of independent explanators of mean temporal muscle thickness.

	**Standardized coefficients^†^**	***P*-value**
Grip strength	0.634	0.000
SARC-F	−0.181	0.012
Age	−0.200	0.015
Sex	−0.425	0.000
BMI	−0.027	0.646
GNRI	0.108	0.084
CCI	0.069	0.406
	*R^2^* = 0.737

† β value.

Furthermore, as shown in Table 3, the mean TMT differed significantly between good and poor initial functional states after stroke (odds ratio: 2.18; *P* < 0.05). The days measured from stroke onset to K-MBI evaluation in two groups were as following: poor function group = 2.83 days (SD ± 2.05), good function group = 3.16 days (SD ± 1.62) (*P* > 0.5).

**Table 3:** Logistic regression analysis for initial functional state after stroke per the K-MBI score.

**Independent variables**	**Adjusted odds ratio (95% CI)**	***P*-value**
TMT	2.18 (1.20–3.94)	0.01
Age	1.03 (0.97–1.11)	0.31
Sex	12.4 (2.27–67.28)	0.04
BMI	0.98 (0.82–1.17)	0.78
GNRI	0.96 (0.89–1.03)	0.27
CCI	1.07 (0.72–1.60)	0.74

## 4 Discussion

This study aimed to suggest TMT as a possible screening marker of pre-stroke sarcopenia in patients with acute ischemic stroke as a substitute of grip strength measurement. Our study suggests the mean TMT as an independent explanator, that is highly associated with grip strength in patients with an initial acute stroke.

TMT measurements could be easily performed on patients, regardless of their cognitive decline. Also, as brain MRI is the gold standard diagnostic technique for acute ischemic stroke, it does not require additional costs. Furthermore, as it is measured from brain imaging upon arrival at the emergency department in our hospital, this could easily reflect the pre-stroke status. Katsuki et al. (33) first proposed the mean TMT measured via CT as an indicator of the premorbid activity of daily living in patients with acute subarachnoid hemorrhage. Steindl et al. demonstrated a moderate relationship between TMT and hand grip strength among patients with cerebrovascular diseases. In this study, as fewer than 30 patients were enrolled in the cerebrovascular disease subgroup, the correlation between the two parameters might have weakened (19). One study reported that standard values of TMT for healthy patients did not differ between Japanese groups and Caucasian groups (28). However, as there is a difference in skeletal muscle mass between Caucasian and Asian groups; therefore, studies on the correlation between TMT and hand grip strength in Asian groups are required. Furthermore, our study focused on the relationship between TMT and grip strength in the acute ischemic stroke group, which was not yet previously studied.

Manual TMT measurement revealed high reliability, just as seen in previous studies (19, 28, 29, 34). The reliability of TMT measurement in numerous studies suggests that this method could be applied in clinical settings. Brain MRI, which was chosen as our tool for measurement, was more accurate than CT in the measurement of the temporal muscle.

SARC-F is a validated useful questionnaire in sarcopenia screening (35, 36). Nozoe et al. (37) suggested that TMT and temporal muscle area measured via CT were associated with the initial sarcopenia risk assessed using the SARC-F questionnaire in patients with acute stroke. Our study also showed the mean TMT measured via MRI was negatively associated with SARC-F score. Compared to this study, our logistic linear regression model was more explanatory, as the *R*^2^ value was 0.737. Furthermore, the inclusion criteria of previous studies were restricted to patients who could walk, and had high cognition levels to cooperate with questionnaires. This may bring accuracy to questionnaires; however, it makes it difficult to represent general stroke patients. In our study, the ability to walk and high cognitive function were not the required criteria for inclusion. Instead, we only included patients who scored a maximum of 14 points within the hand function criteria of the Fugl-Myer assessment of the non-hemiplegic hand. This was included to obtain credentials for maximal grip strength measurement. Scoring maximum points in hand function criteria of the Fugl-Myer assessment directly shows that the patient can fully perform the non-hemiplegic hand's function, although the patient might have other disabilities. This allowed our study to be more representative of the stroke population with unilateral hemiplegia. Even in this different eligibility criteria settings, our study also suggests TMT as a predictive factor for sarcopenia risk.

In addition, we suggest that TMT might be related to the functional level of initial stroke patients. Logistic regression analyses revealed that TMT was associated with the initial status of activity of daily living after stroke, which was measured using the K-MBI score. Time from stroke onset to functional assessment showed no evidence of difference between the two groups with different functional ability. In a previous study, Nozoe et al. (38) suggested that the initial stroke severity measured by National Institutes of Health Stroke Scale (NIHSS) was associated with lower extremity muscle strength within the first 5 days after stroke onset in older patients. As numerous studies suggest that TMT is closely related to one's skeletal muscle status, we could expect that functional dependency at initial acute stroke phase might be predicted by the TMT value. Further studies are required to assess the relationship of TMT and stroke severity in acute phase.

Our study has several limitations. First, due to our hospital's MRI protocol, we took FLAIR images to measure TMT. Previous studies measured TMT with both T1 and T2 images, while there are more ones with T1, as it makes it easier to measure TMT manually. The measurement process needed additional tracing of images to see the accurate temporal muscle border. However, as both inter-rater, and intra-rater reliability were high, we could see that each measurement's accuracy remains. Second, we did not investigate factors that could focally affect TMT regardless of generalized sarcopenia, such as cranio-bulbar diseases or orthodontic problems that could lead to muscle atrophy or edema (39, 40). Third, the small number of patients were enrolled, with relatively few female patients. A sample size of 103 was required for power of 0.8, an alpha error of 0.05, a degree of freedom of 7, and a medium effect size (ω = 0.15), but it was yet fulfilled in this study. The enrollment of more patients could strengthen the statistical power of this study. Longitudinal assessments of the relationship between TMT and grip strength, including the subacute and chronic stroke phases, should be further studied. Therefore, research on the association between initial TMT and functional outcomes during the post-stroke period should be done to establish the clinical significance of TMT.

In conclusion, TMT of acute ischemic hemiplegic stroke patients showed a strong correlation with the grip strength of the unaffected arm. Measuring TMT using cranial MR images at the initial stage of stroke could help predict a patient's pre-stroke muscle strength status. Further studies on the application of TMT in the diagnosis of pre-stroke sarcopenia are required.

## Data availability statement

The original contributions presented in the study are included in the article/Supplementary material, further inquiries can be directed to the corresponding author.

## Ethics statement

The studies involving humans were approved by Institutional Review Board of Eunpyeong St. Mary's Hospital (IRB PC22RASI0242). The studies were conducted in accordance with the local legislation and institutional requirements. Written informed consent for participation was not required from the participants or the participants' legal guardians/next of kin in accordance with the national legislation and institutional requirements.

## Author contributions

Conceptualization and project administration: JihP. Methodology, validation, and writing—review and editing: JisP and JihP. Formal analysis: JisP, SK, and DK. Investigation: JisP, SK, DK, and JihP. Data curation: JisP and SK. Writing—original draft preparation: JisP. All authors contributed to manuscript revision, read, and approved the submitted version.
