# Supplementary material for: Correlation between temporal muscle thickness and grip strength in hemiplegic patients with acute stroke
Source: Front Neurol. 2023 Nov 23;14:1252707. doi: 10.3389/fneur.2023.1252707 (PMC10701424; doi:10.3389/fneur.2023.1252707)
Supplement: Supplementary file 1 [file Data_Sheet_1.PDF]

NO	SEX	AGE	GNRI	CCI	SARC-F	BMI	TMT	K-MBI	GRIP - Unaffected hand
1	F	78	104.1986	5	1	0.002308	6.465	39	18.9
2	M	61	112.5564	7	0	0.002348	11.045	79	33
3	F	50	110.922	4	0	0.002281	9.77	76	22
4	M	60	106.7259	8	0	0.002323	11.9	76	28
5	M	81	110.1975	6	0	0.00264	9.4	41	28
6	M	67	114.4417	4	0	0.00254	12.2	32	38
7	M	62	111.4773	4	0	0.002318	11.895	55	37
8	M	54	97.04358	3	0	0.001913	9.82	38	30
9	F	60	83.27915	9	4	0.001543	6.68	18	12
10	F	75	112.1783	8	0	0.002424	8.905	20	16
11	F	90	109.0457	7	5	0.002289	6.045	19	10
12	M	58	109.9914	3	0	0.002438	10.8	57	32
13	M	47	114.6806	3	0	0.002665	11.26	19	33
14	M	53	123.3296	3	0	0.003071	12.035	86	33
15	M	86	107.3967	7	0	0.00215	8.565	30	31
16	F	78	113.3714	6	8	0.002444	6.615	8	16
17	M	79	105.2165	13	4	0.002374	8.93	55	25.8
18	F	72	112.2841	6	0	0.002592	8.26	75	17
19	M	63	105.5627	5	0	0.002126	10.14	67	24
20	F	65	122.8784	6	4	0.002585	9.64	61	23
21	M	35	109.0043	2	1	0.002232	9.41	26	20
22	M	55	90.62352	4	0	0.002028	11.03	32	35.5
23	F	72	108.7192	5	0	0.002019	8.925	61	22.8
24	M	80	97.01703	9	1	0.002145	8.22	38	27
25	M	64	109.7418	4	0	0.002606	10.51	13	29.5
26	M	64	106.6238	4	0	0.001957	10.83	16	35
27	F	51	95.56612	3	0	0.00208	8.65	13	14
28	M	57	129.5965	3	0	0.003144	12.665	26	37

29	M	66	116.9718	4	0	0.002657	9.34	54	33
30	M	78	91.88104	7	0	0.001754	8.05	58	22
31	F	88	102.7688	6	2	0.002081	8.8	23	14
32	M	85	102.6318	9	4	0.002192	8.505	9	25.7
33	M	83	117.0824	7	1	0.002381	9.78	56	32.5
34	M	77	91.16073	8	2	0.001917	10.165	0	22
35	M	93	96.14873	6	5	0.002199	7.34	5	19.5
36	M	78	115.3313	6	0	0.002681	10.58	31	23.3
37	F	62	112.4666	6	0	0.002289	9.705	90	21.2
38	M	78	106.6813	7	2	0.002422	10.71	28	27.3
39	F	72	106.5762	5	5	0.002121	6.46	12	10
40	F	65	101.613	5	2	0.002556	9.1	47	17.5
41	M	57	108.3572	4	0	0.002571	12.71	45	39.2
42	F	75	118.9697	6	4	0.002622	6.475	57	13
43	F	73	115.4524	5	0	0.002604	9.195	55	21
44	F	82	106.9579	7	2	0.002458	8.15	76	10
45	F	77	99.82738	8	5	0.002	5.4	28	8
46	F	85	111.224	7	4	0.002362	6.8	13	15
47	M	61	112.7823	6	3	0.002444	11.835	57	38.5
48	F	83	105.2967	6	2	0.002081	8.085	14	14.5
49	M	71	97.62479	6	0	0.00209	7.845	71	18.6
50	M	59	103.7091	4	0	0.002028	11.425	48	35
51	M	64	104.9184	4	0	0.002243	10.635	80	44.9
52	F	81	109.9335	6	1	0.002728	6.91	5	10
53	M	49	112.8344	3	0	0.002571	11.695	100	29.5
54	F	85	107.3513	7	2	0.002003	8.405	13	12.7
55	F	37	106.8526	2	0	0.002094	9.845	77	35.2
56	F	68	121.7847	3	0	0.00253	9.135	27	3.5
57	M	78	100.4149	7	0	0.002161	8.56	82	29.4

58	M	91	107.2398	7	2	0.002231	7.55	40	20.4
59	F	83	112.2841	8	3	0.002264	8.14	53	22.8
60	M	65	108.0491	4	0	0.002342	9.095	79	33.9
61	M	57	112.3883	5	0	0.002571	10.34	81	25.5
62	M	76	120.9258	6	0	0.002743	11.485	85	33.6
63	M	85	103.1441	7	1	0.00213	8.725	100	24.5
64	M	81	101.658	6	3	0.002081	9.035	83	23.3
65	M	64	116.8171	4	0	0.002574	10.335	8	29.5
66	M	66	108.3525	4	0	0.002042	9.025	82	30.5
67	M	59	112.9273	3	0	0.002286	8.95	61	23.5
68	M	75	102.8661	6	2	0.002637	7.995	49	25.5
69	F	80	124.1483	7	1	0.003205	7.855	45	15.1
70	M	74	110.1955	7	0	0.002346	11.215	68	30.2
71	M	53	101.7748	3	0	0.002286	9.97	32	31.2
72	M	62	99.18044	4	2	0.001937	11.62	20	30.4
73	M	67	110.1007	4	0	0.002561	11.46	10	29.9
74	F	70	103.0506	5	0	0.001992	8.525	84	16.8
75	M	63	114.9185	6	0	0.002559	9.29	68	26.1
76	F	72	117.86	8	0	0.002819	8.855	20	17.7
77	M	67	114.4868	4	0	0.002704	11.71	29	43.3
78	F	73	114.0605	5	4	0.002584	8.15	46	17.1
79	M	71	108.0496	5	0	0.002283	9.02	43	21.5
80	F	51	105.7897	3	0	0.002094	8.32	7	17.7
81	M	33	121.2108	2	0	0.002673	10.355	63	39.5
82	M	56	117.0778	4	0	0.002893	11.115	60	30.1
83	F	36	130.224	2	0	0.003151	11.005	35	27.7
84	F	70	95.2448	6	2	0.002434	8.23	15	15.5
